# Mechanism of Coupling Twist Angle and Projectile Radius on Ballistic Impact Performance of Bilayer Phosphorene Membranes

**DOI:** 10.3390/nano15181414

**Published:** 2025-09-14

**Authors:** Ning Liu, Ke Huang, Xuejian Yang, Dongdong Xu, Lihua Wang

**Affiliations:** 1 School of Aerospace Engineering and Applied Mechanics, Tongji University, Shanghai 200092, China; 2 Shanghai Institute of Aircraft Mechanics and Control, 100 Zhangwu Road, Yangpu District, Shanghai 200092, China; 3 School of Mechanical Engineering, Tongji University, Shanghai 201804, China

**Keywords:** twisted phosphorene bilayer, ballistic impact, cone wave, coarse-grained modeling

## Abstract

This study investigated the coupling mechanism between interlayer twist angle and projectile size on the ballistic performance of bilayer phosphorene membranes, a topic essential for designing efficient nano-protective materials, yet still poorly understood. Using coarse-grained molecular dynamic simulations, we systematically explored how twist angles (0–90°) and projectile radii (2–10 nm) jointly influence impact response for membranes with a radius equal to 48 nm. We found that the effect of twist angle becomes significant only beyond a critical projectile size (~8 nm). Below this threshold, deformation remains local and twist-independent. However, for larger projectiles, the twist angle drastically alters wave propagation and failure modes. Specifically, a 90° twist induces severe wave reflection and interference, leading to a dramatic force amplification (up to 82%) and a 28% reduction in ballistic limit velocity, making it the most susceptible configuration. These results underline the critical role of twist–boundary–wave interaction in governing impact resistance and provide practical insights for the design of phosphorene-based nano-armor systems tailored to specific impact conditions.

## 1. Introduction

Two-dimensional (2D) materials, such as phosphorene, with its distinctive puckered lattice and pronounced mechanical anisotropy [1,2,3], exhibit exceptional stiffness, strength, and capacity to delocalize impact energy, making them highly promising for high-velocity impact protection [2,4,5,6,7,8]. In bilayer configurations, the relative twist angle between layers generates complex moiré superlattices, significantly influencing interfacial interactions and mechanical responses [3,9,10,11]. However, the intrinsic nanoscale features of such materials induce size-dependent behaviors distinct from macroscale counterparts [12,13,14,15,16,17,18]. Compounding this complexity, experimental challenges in probing nanoscale impact dynamics—particularly energy dissipation mechanisms under supersonic conditions [19,20]—obscure the impact protection capabilities of 2D nanostructures like twisted bilayer phosphorene [21,22,23]. The structure–property relationships governing high-velocity impacts are further complicated by strain-rate sensitivity [23,24], length-scale effects related to specimen dimensions and projectile radius [25,26,27], geometric nonlinearity [4,28], and phosphorene’s inherent anisotropy [1,2,3]. Crucially, the coupled effects of the interlayer twist angle and projectile radius on the ballistic impact performance of bilayer phosphorene remain poorly understood, representing a significant knowledge gap. Consequently, a rigorous understanding of these relationships is imperative for designing high-performance phosphorene-based protective shields [29,30,31]. Coarse-grained (CG) molecular dynamic simulations, overcoming the spatiotemporal limitations of both experiments and all-atom MD, emerge as an essential tool to unravel nanoscale ballistic impact mechanisms, specifically enabling the systematic investigation of the synergistic coupling between twist angle and projectile radius in bilayer phosphorene membranes.

The introduction of a twist angle between adjacent layers in two-dimensional (2D) materials has emerged as a powerful tool for engineering quantum electronic, thermal, and mechanical properties, primarily through the formation of moiré superlattices that impose periodic potentials and reconfigure interlayer coupling dynamics [3,9,10,11,32,33,34,35,36,37]. In twisted bilayers, the moiré angle directly dictates the periodicity and symmetry of these superlattices, enabling unprecedented modulation of phenomena such as bandgap tuning in transition metal dichalcogenides (e.g., ReS_2_, where a 0–10° twist adjusts exciton energy by 40 meV) [32], thermal conductivity in graphene (78% reduction under strong interlayer coupling), and phonon transport in puckered materials like phosphorene, where a “phonon magic angle” preserves or enhances thermal conductivity due to van der Waals confinement and minimized anharmonicity [33].

Specifically, phosphorene’s in-plane anisotropy and puckered lattice amplify the twist-angle sensitivity, as demonstrated in 90 degree-twisted bilayers where overlapping regions develop distinct band structures that drastically alter carrier transport under external fields [38]. This tunability mirrors the hierarchical reinforcement mechanism of Bouligand structures [10,11,39,40]—biologically inspired architectures where layered fibrils are rotated incrementally to deflect cracks and dissipate impact energy. While Bouligand designs exploit multi-angular interfaces to optimize mechanical resilience [11], analogous principles may govern twisted 2D membranes, where moiré-induced strain redistribution and interlayer decoupling could critically influence dynamic failure modes under ballistic loading. With respect to ballistic loading, 2D materials like graphene shows specific penetration energies an order of magnitude higher than macroscopic steel sheets [41]. This performance stems from stress delocalization during failure, where tensile implosion waves generate conical fronts that propagate radially, forming petal-like cracks [41]. The ultra-thin 2D material membrane’s deformation around these expanding cones redistributes kinetic energy over larger areas, underpinning 2D materials’ superior dissipation capacity. However, in phosphorene—with its inherent puckered structure and strong mechanical anisotropy [1,2,3]—cone wave dynamics differ fundamentally. The finite specimen size causes cone wave reflections that concentrate strain catastrophically [41,42], while phosphorene’s high specific stiffness accelerates wave propagation, even under supersonic impacts [4]. Specifically, the mechanical anisotropy of phosphorene dictate the shape of the cone wave front and consequently affect the cone wave reflection and the associated strain concentration [42,43,44]. Due to the finite size of specimens, cone wave reflection can cause significant degradation in the impact resistance of phosphorene and phosphorene-based nanostructures. Crucially, this degradation exhibits pronounced size dependency, where geometrical nonlinearity, intrinsic anisotropy, and—in bilayer systems—moiré patterns induced by interlayer twist angles modulate strain localization and wave reflection paths. The synergistic coupling between twist angle and projectile radius thus emerges as a pivotal, yet unexplored factor governing cone wave dynamics and ultimate failure in bilayer phosphorene membranes.

Coarse-grained (CG) modeling has emerged as a pivotal methodology for probing nanoscale impact phenomena in 2D materials, balancing computational efficiency with the capacity to capture multi-scale behaviors inherent to systems like anisotropic puckered phosphorene [24,25,45,46,47]. While atomistic molecular dynamic (MD) simulations have elucidated failure mechanisms in 2D materials under ballistic impact [4,22,48,49,50], they are typically constrained to nanoscale projectiles (several nanometers) to manage computational costs. This limitation confines observations to localized failure processes that may inadequately represent bulk material responses, particularly for larger projectiles relevant to practical impact scenarios. Furthermore, atomistic approaches often rely on computationally intensive bond-order potentials to model bond rupture [51,52,53,54], despite ballistic impacts occurring over timescales too short for significant bond reconfiguration [55]. For bilayer phosphorene—where interfacial moiré patterns induced by twist angles demand large-scale models—CG MD becomes essential. By extending spatiotemporal scales beyond atomistic MD, CG simulations uniquely enable systematic investigation of size-dependent failure governed by projectile radius while concurrently resolving how twist angle-modulated interfacial mechanics influence energy dissipation and wave propagation. This approach is indispensable for unraveling the synergistic coupling between twist angle and projectile radius in phosphorene membranes under high-velocity impact.

Here, CG MD simulations were utilized to simulate dynamic responses of nanoscale bilayer phosphorene membranes under the ballistic impact of diamond projectiles. We also studied the effect of twist angle and projectile radius on impact behaviors of phosphorene membranes, in which the role of the cone wave reflection and interference was emphasized in the length-scale dependence of ballistic limit reduction for twisted bilayer phosphorene membranes.

## 2. Materials and Methods

**Figure 1:**
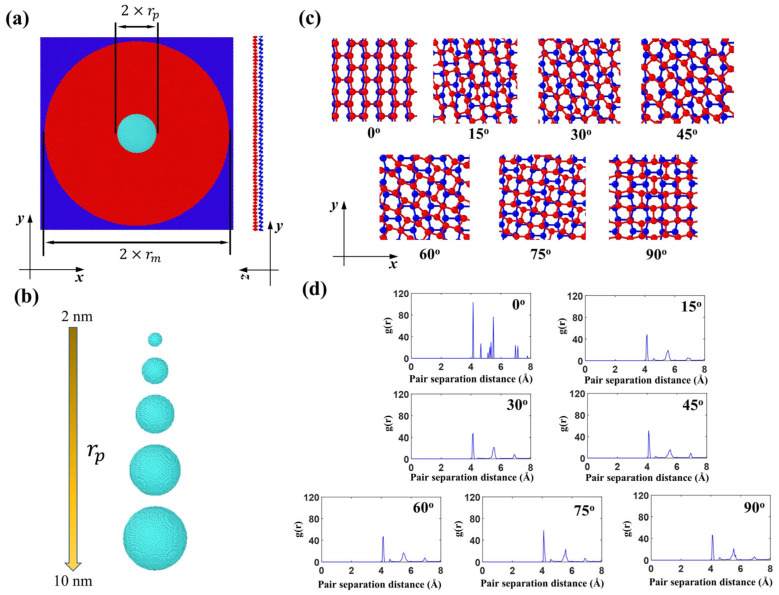
Coarse-grained (CG) model for the impact of bilayer phosphorene membranes by diamond projectiles. (**a**) Overview of the CG model of the bilayer phosphorene membrane and the spherical diamond projectile; (**b**) snapshot of the diamond projectiles with radius ranging from 2 to 10 nm; (**c**) top view of the bilayer phosphorene membranes with different twist angles; (**d**) radial distribution function of the bilayer phosphorene sheets with different twist angles.

As shown in Figure 1, the impact performance of bilayer phosphorene membranes was studied under the shock of diamond projectiles. Figure 1a shows the top view of the simulation setup, in which the blue edges of the bilayer phosphorene membrane are fixed, while the red circular center is free to move with radius $r_{m}$ = 48 nm.

A diamond projectile, modeled as a rigid body based on previous laser-induced projectile impact tests showing negligible deformation during high-velocity impact [41], was positioned centrally above the bilayer phosphorene membrane. This rigid-body assumption was further validated by prior atomistic simulations demonstrating imperceptible diamond deformation under Tersoff potential [55]. An initial 10.5 nm vertical separation prevented pre-impact interactions. The projectile was represented by CG beads arranged in a diamond cubic lattice (lattice constant = 0.72 nm), providing a simplified, yet structurally consistent representation at twice diamond’s atomic lattice spacing. Each CG bead had a mass of 96 g/mol, yielding a bulk density of 3.42 g/cm^3^, closely approximating crystalline diamond (3.5 g/cm^3^) [42]. Projectile radii ranged from 2 to 10 nm (Figure 1b). Bilayer phosphorene membranes were constructed using an established CG model [7,56,57], with the bottom layer fixed and the top layer rotated to achieve twist angles θ = 0–90° (Figure 1c). Radial distribution function (RDF) analysis (Figure 1d) revealed distinct structural evolution: at θ = 0°, sharp multiple peaks indicated high intralayer and interlayer crystalline order. Progressive twisting (15–90°) induced interlayer distortion through strong inter-sheet adhesion, manifested as peak broadening and reduced peak count.

All MD simulations were conducted within the LAMMPS computational framework [58], with atomic configurations visualized using OVITO [59]. This integrated workflow enabled high-fidelity tracking of membrane deformation and penetration dynamics during ballistic events. The selection of the time step (Δt) is critical for numerical stability and physical accuracy, particularly for capturing the fastest vibrational modes in the system—specifically CG bead interactions modeled as harmonic oscillators. The governing equation $\omega =\frac{2\pi }{T}=\sqrt{\frac{k}{m}}\;$ defines the angular frequency, where k represents bond stiffness and m the CG bead mass. To ensure precise temporal resolution of these vibrations, we maintained Δt<T/10 ($T\;=\frac{2\pi }{\omega }$ being the oscillation period), resulting in a theoretical upper limit of ∼33 ps for phosphorene systems. All simulations employed a conservative Δt = 1 fs throughout this work, providing 33× higher resolution than the stability threshold. This rigorous temporal discretization guarantees accurate resolution of bond fracture dynamics during high-velocity impacts while maintaining computational feasibility.

Projectile–membrane interactions were governed by a 12-6 Lennard-Jones potential with parameters $\varepsilon_{LJ}$ = 0.167 eV and $r_{LJ}$ = 4.45 Å [7]. Prior computational studies confirmed these parameters negligibly influence ballistic response metrics: force profiles and velocity evolution remain consistent across varying $\varepsilon_{LJ}$ values [7,42,56]. While increased cohesive energy elevates force measurement variance, mean values show minimal deviation [7,42,56]. Phosphorene membranes underwent 10 ps NVT equilibration at 10 K to suppress thermal noise [56]. Impact simulations initialized projectiles with velocities perpendicular to the membrane plane, with subsequent dynamics computed under NVE ensemble conditions to maintain energy conservation, following established impact protocols [1,4,27,42].

Particle trajectories for both projectiles and phosphorene membranes were sampled at 0.5 ps intervals. These time-resolved data enabled computational derivation of projectile acceleration $a_{p}(t)$ and impact force $F_{p}(t)$ employing a central difference scheme:

(1) $$a_{p}(t)=\frac{V\left(t+\Delta t\right)-V(t-\Delta t)}{2\Delta t}$$

(2) $$F_{p}(t)=m_{p}a_{p}(t)$$

where Δt= 0.5 ps denotes the output interval, V(.) represents instantaneous projectile velocity, and $m_{p}$ is total projectile mass. The acceleration profile was thus computed from velocity differentials across consecutive time steps.

## 3. Results

### 3.1. Effect of Twist Angle on Impact Performance

**Figure 2:**
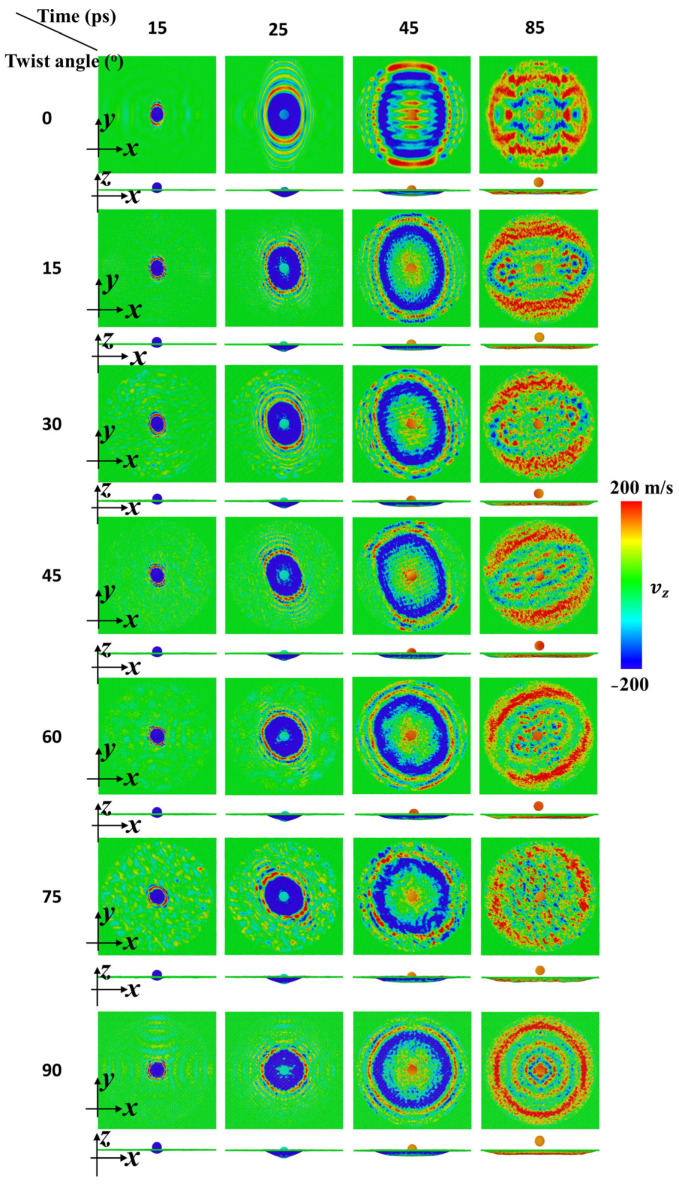
Velocity contours of the bilayer phosphorene membranes with different twist angles during impact of a diamond projectile with radius $r_{p}$ = 4 nm (initial velocity of the projectile, $V_{0}$, is 800 m/s).

In order to investigate the effect of twist angle on impact performance, CG models were applied to simulate ballistic impact of bilayer phosphorene membranes, in which the twist angle ranged from 0° to 90° while the radius of the projectile $r_{p}$ was 4 nm. As shown in Figure 2, the impact dynamics of the projectile, with its initial velocity $V_{0}$ equal to 800 m/s, and the bilayer phosphorene are reflected by the simulation snapshots colored by the velocity of CG beads in both phosphorene membranes and projectiles. For each twist angle, four snapshots were taken. Initially, the projectile fell at a constant velocity until it collided with the phosphorene membrane. After the collision, the phosphorene membrane was pushed down while a blue dot formed at the collision center of the phosphorene membrane, as shown in the first column of the snapshots at 15 ps in Figure 2. That blue dot is the place where the cone wave initializes [7,42,56].

Later on, the projectile keeps pushing the phosphorene membrane down while the cone wave propagates towards the fixed edge. Consequently, the blue dot grows into a blue ellipse, as shown in the second column of the snapshots at 25 ps in Figure 2. Interestingly, the shape of the blue region changes from ellipse-like to circle-like. According to previous studies [4,21,22,23,41,42,50,56], the propagation speed of the cone wave along a specific direction depends on the elastic modulus along that direction. For phosphorene [7], the elastic modulus along the armchair direction, the x direction of the bottom phosphorene sheet, is 136.7 GPa while that along the zigzag direction, the y direction of the bottom phosphorene sheet, is 34.0 GPa. Consequently, the propagation speed of the cone wave along the armchair direction is faster than that along the zigzag direction for phosphorene. Therefore, when the twist angle is equal to 0°, the shape of the cone wave front is an ellipse with its major axis along the y direction and its minor axis along the x direction. Due to the strong adhesion between the phosphorene sheets, the bottom and top phosphorene sheets deform without slippage or delamination. Furthermore, the global elastic tensor of the bilayer phosphorene membrane evolves in the manner of the arithmetic mean of the elastic modulus for both the bottom and top phosphorene sheet. Accordingly, as the twist angle increases, the shape of the cone wave front alters, in which the major axis rotates counterclockwise and the axial ratio keeps decreasing to 1.0.

Subsequently, the cone wave further propagates while the size of the cone wave front becomes larger, as shown in the third column of the snapshots at 45 ps in Figure 2. Moreover, the cone wave along the major axis travels faster and first reaches the fixed edge. Upon that, the cone wave reflects and interferes with the propagating cone wave, indicated by cross-shaped interference stripes in the third column of Figure 2. Note that inside the cone wave region, the color becomes yellow, indicating the rebounding of the phosphorene membrane. Simultaneously, the projectile moves upwards under the support of the phosphorene membrane. Finally, the cone wave along the minor axis also reflects and interferes with the propagating cone wave, generating two interference centers and thus weakening the strain amplification accordingly. Note that as the twist angle increases from 0° to 90°, the two interference centers also rotate counterclockwise and move towards each other, merging into one for a twist angle equal to 90°. Simultaneously, the projectile detaches from the phosphorene membrane and moves away from the membrane.

**Figure 3:**
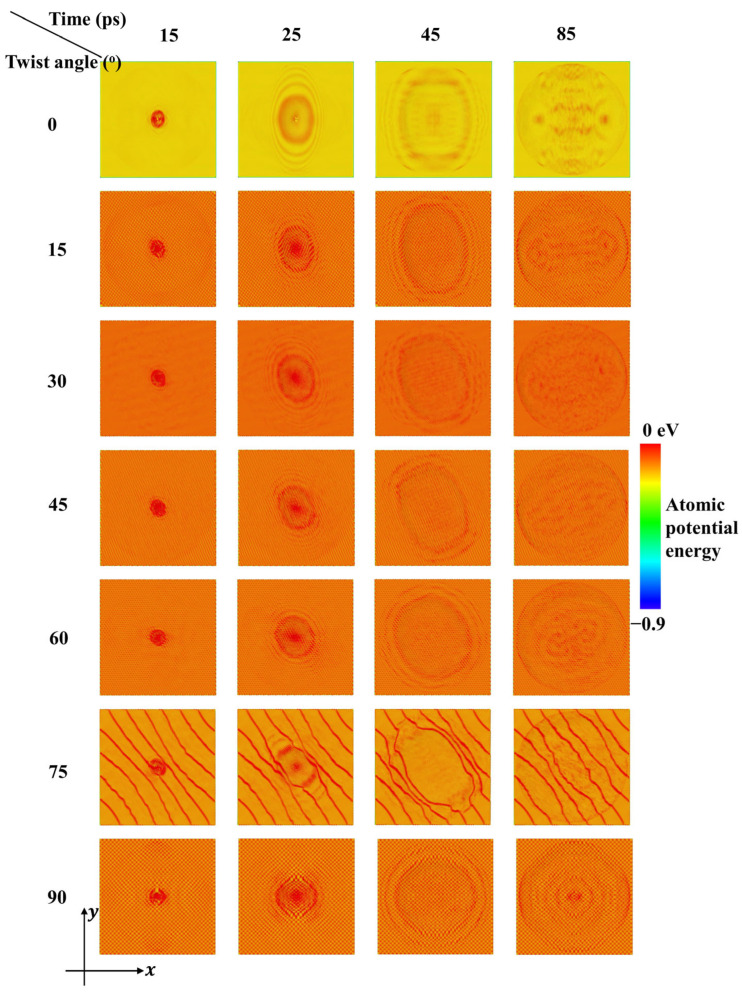
Potential energy contours of the bilayer phosphorene membranes with different twist angles during impact of a diamond projectile with radius $r_{p}$ = 4 nm (initial velocity of the projectile, $V_{0}$, is 800 m/s).

**Figure 4:**
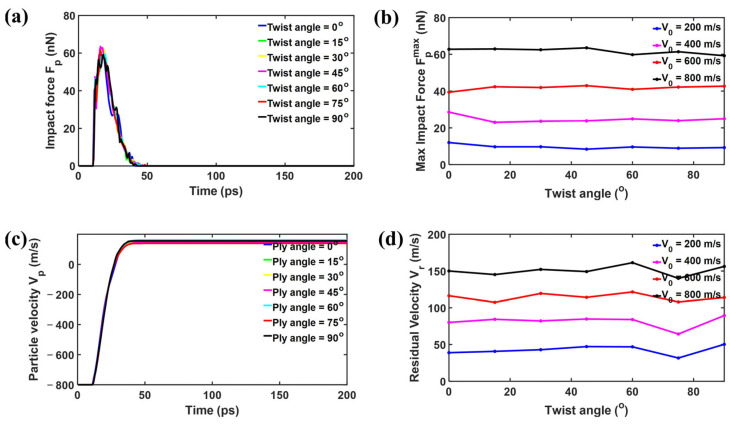
Impact dynamics of the diamond projectile with radius $r_{p}$ = 4 nm upon bilayer phosphorene membranes with different twist angles: (**a**) the impact force $F_{p}$ profiles of the diamond projectile when the initial velocity $V_{0}$ = 800 m/s; (**b**) maximum impact force ${F_{p}}^{max}$ versus twist angle; (**c**) the projectile velocity $V_{p}$ profiles of the diamond projectile when the initial velocity $V_{0}$ = 800 m/s; (**d**) residual velocity $V_{r}$ versus twist angle.

Figure 3 shows the atomic potential energy contours for bilayer phosphorene membranes with different twist angles, in which bond energy, angle energy, dihedral energy, and non-bonded pair energy are all included. The variation trends of atomic potential energy are very similar to the atomic velocity contours in Figure 2. However, it can be seen that the baseline of potential energy for membranes with different twist angles are different, as reflected by the background color. The background color for 0 degrees is yellow, that for 75 degrees is light orange, while for the other twist angles the background color is orange. These color configurations indicate that degree 0 has the lowest potential energy baseline, degree 75 has the second-lowest potential energy baseline, while the potential energy baselines for the other twist angles are very close to each other and thus significantly higher than the aforementioned two degrees. For degrees 15–60 and 90, there are dense diagonal checkered stripes, while the orientation rotates counterclockwise as the twist angle degree increases. In contrast, for degree 0, there are no such stripes, which indicates the low level of tortuosity. These differences in stripe patterns are in good agreement with the peak patterns of radial distribution function in Figure 1d, in which sharp peaks indicate high intralayer and interlayer crystalline order for degree 0, while blunt peaks indicate distortion and deformation for the other degrees. More interestingly, for degree 75, there are sparse diagonal stripes, which are significantly different from degrees 15–60 and 90. This sudden change in geometric configuration and energy landscape leads to an abrupt decrease in residual velocity in Figure 4, which is discussed later.

For the goal of further exploring the force changes during the impact between the projectile and the bilayer phosphorene membrane, the force–time curves for a certain initial velocity of the projectile, $V_{0}$ = 800 m/s, are shown in Figure 4a. The whole process can be divided into three stages. In stage I, the impact force $F_{p}$ remains zero. In this stage, the projectile moves towards the phosphorene membrane, while the distance between them is way larger than the cutoff for force calculation, resulting in the zero-force responses. In stage II, the impact force $F_{p}$ quickly increases as the projectile pushes the membrane down. After reaching the peak, the impact force $F_{p}$ decreases as the projectile velocity further decreases and starts to increase. During this stage, the projectile first pushes the membrane down and then bounces back under the support of the membrane. In stage III, the impact force becomes zero again as the projectile detaches and then flies away from the membrane. Interestingly, the impact force $F_{p}$ almost coincides with the different twist angles. To further test the effect of twist angle on maximum impact force, additional simulations with different initial velocities of projectiles, namely $V_{0}$, were performed. Figure 4b shows the maximum impact force ${F_{p}}^{max}$ versus twist angle, in which ${F_{p}}^{max}$ barely changes as the twist angle alters. As the initial velocity $V_{0}$ changes from 200 to 800 m/s, the maximum impact force increases, while for a specific $V_{0}$, the maximum impact force ${F_{p}}^{max}$ remains almost the same for different twist angles. Figure 4c shows the projectile velocity evolution with time during impact with the initial velocity $V_{0}$ fixed at 800 m/s. Similarly to the impact force, the projectile velocity almost coincides with different twist angles in association with time. The initial velocity $V_{0}$ is 800 m/s, while the residual velocity $V_{r}$ fluctuates around 150 m/s, indicating a significantly high energy absorption rate. To further test the effect of twist angle on residual velocity, additional simulations with different initial velocities of projectiles, namely $V_{0}$, were performed. Figure 4d shows the residual velocity $V_{r}$ versus twist angle, in which $V_{r}$ barely changes as the twist angle alters. As the initial velocity $V_{0}$ changes from 200 to 800 m/s, the residual velocity $V_{r}$ increases, while for a specific $V_{0}$, the residual velocity $V_{r}$ remains almost the same for different twist angles.

**Figure 5:**
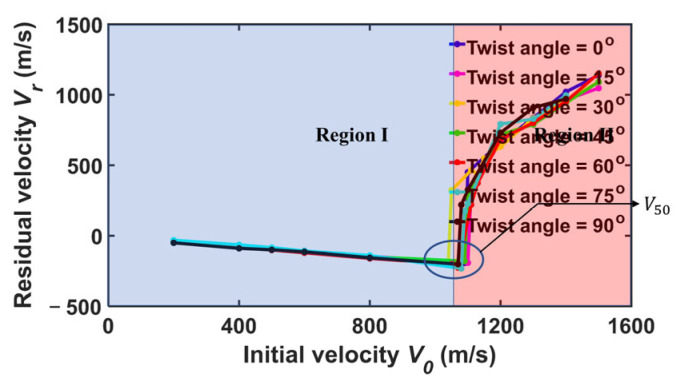
Residual velocity $V_{r}$ versus initial velocity $V_{0}$ of the diamond projectile with radius $r_{p}$ = 4 nm upon bilayer phosphorene membranes with different twist angles.

**Figure 6:**
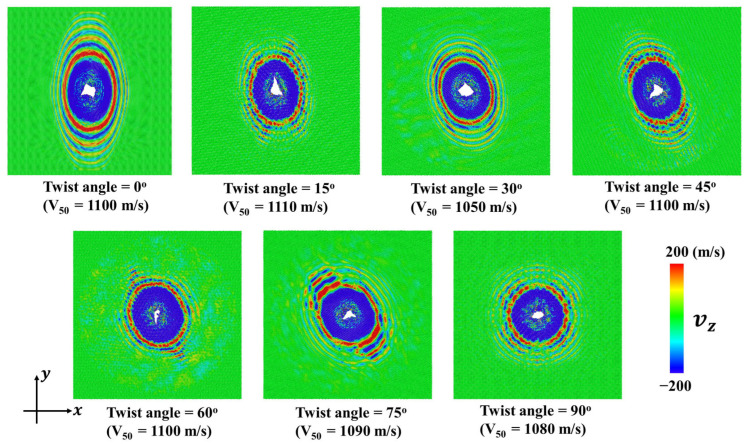
Fracture patterns of the bilayer phosphorene membranes with different twist angles under the impact of the diamond projectile with radius $r_{p}$ = 4 nm when the initial velocity $V_{0}$ is equal to the ballistic limit $V_{50}$.

Figure 5 shows the residual velocity $V_{r}$ versus initial velocity $V_{0}$ for the diamond projectile with $r_{p}$ = 4 nm upon bilayer phosphorene membranes with different twist angles. It can be seen that the curves can be divided roughly into two regions. Region I represents the scenario where the projectile cannot penetrate the membrane and bounce back. Moreover, in this region, the residual velocity $V_{r}$ changes linearly with the initial velocity $V_{0}$. Region II represents the scenario where the projectile can penetrate the membrane far before the cone wave reaches the fixed boundary, in which the failure is local instead of global. It can be seen that the variation of residual velocity $V_{r}$ versus initial velocity $V_{0}$ are almost the same for different twist angles.Note that the critical velocity beyond which the projectile can penetrate the bilayer phosphorene membrane is the ballistic limit, usually denoted $V_{50}$ [42,56]. Here, for the diamond projectile with $r_{p}$ = 4 nm, the ballistic limit $V_{50}$ is around 1100 m/s regardless of the twist angle for the circular bilayer phosphorene membrane with $r_{m}$ = 48 nm. Figure 6 shows the fracture patterns of the phosphorene membranes under the ballistic limit. It can be seen that although the cone wave fronts are different in shape for different twist angles, structural failure happens before the cone wave reaches the boundary. Moreover, the fracture areas are all located in the collision center, indicating a local failure instead of a global failure.

### 3.2. Effect of Projectile Radius on the Impact Performance

**Figure 7:**
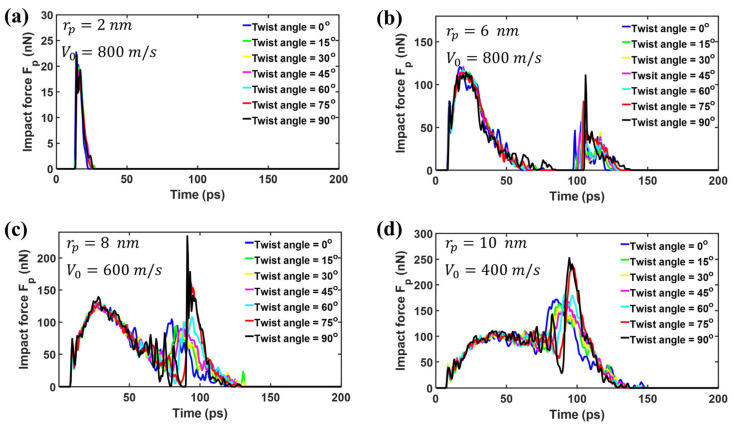
The impact force $F_{p}$ of the diamond projectiles with different $r_{p}$ upon bilayer phosphorene membranes with different twist angles. (**a**) $r_{p}$ = 2 nm, $V_{0}$ = 800 m/s; (**b**) $r_{p}$ = 6 nm, $V_{0}$ = 800 m/s; (**c**) $r_{p}$ = 8 nm, $V_{0}$ = 600 m/s; (**d**) $r_{p}$ = 10 nm, $V_{0}$ = 400 m/s.

In order to investigate the effect of projectile radius on the impact performance, CG models were applied to simulate the ballistic impact of bilayer phosphorene membranes, in which the radius of the projectile $r_{p}$ ranges from 2 to 8 nm. Figure 6 shows the impact force $F_{p}$ versus time. As shown in Figure 7a, the patterns of impact force evolution for $r_{p}$ = 2 nm is very similar to that of $r_{p}$ = 4 nm, as shown in Figure 4a. Specifically, the force curves are spike-like for $r_{p}$ = 2 nm and $r_{p}$ = 4 nm. Interestingly, the force curves are different in shape for $r_{p}$ = 6 nm, as shown in Figure 7b, in which there are two spikes instead of one spike. Similarly to $r_{p}$ < 4nm, the first spike for $r_{p}$ = 6 nm is attributed to the initial impact between the projectile and the phosphorene membrane. In contrast, the second spike in Figure 7b results from the reflection and interference of the cone wave [42,56]. Fortunately, the second spike is not higher than the first spike in Figure 7b. Therefore, structural failure under the impact of projectiles with $r_{p}$ = 6 nm is still dominated by the initial collision between the projectile and the phosphorene membrane. Consequently, the structural failures are still local for $r_{p}$ = 6 nm.

Figure 7c shows the impact force $F_{p}$ of the diamond projectiles with $r_{p}$= 8 nm upon bilayer phosphorene membranes. The whole force responses can be divided into two phases. In phase I, the impact force $F_{p}$ first increases and then decreases. In this phase, the force curves for different twist angles almost coincide with each other, in which there is no reflection and interference for the cone wave. In phase II, the second spike occurs and the curves deviate from each other. Due to different degrees of mismatch between the wave-front shape and the membrane shape, the reflection and interference are also different for bilayer phosphorene membranes with different twist angles. Specifically, the aforementioned mismatch for the twist angle equal to 90° is lowest. Therefore, the force amplification is highest for 90°, as shown in Figure 7c, in which the second spike is significantly higher than the first spike. Hence, the structural failure under the impact of projectiles with $r_{p}$ = 8 nm is dominated by the reflection and interference for the cone wave. As a result, the structural failure mode transitions from local to global. Figure 7d shows the impact force $F_{p}$ for the diamond projectiles with $r_{p}$ = 10 nm. Significantly, the force curve changes further in shape. However, the force curve can still be divided into two phases, in which the first phase is dominated by the initial collision between the projectile and the membrane, while the second phase is dominated by the reflection and interference of the cone wave.

**Figure 8:**
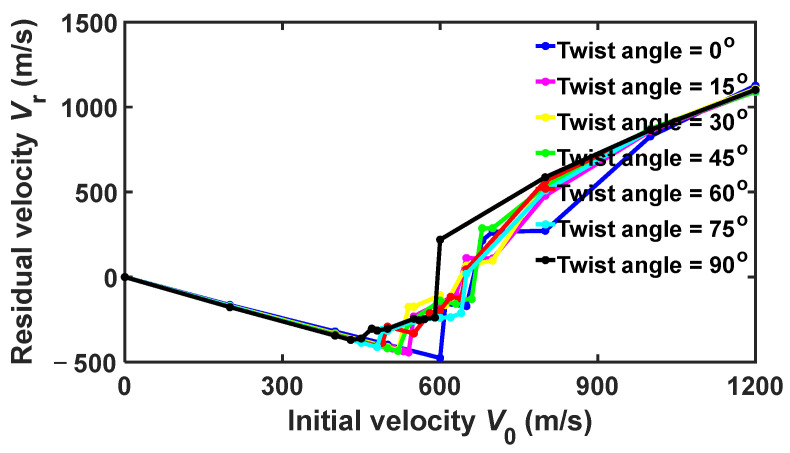
Residual velocity $V_{r}$ versus initial velocity $V_{0}$ of the diamond projectiles with radius $r_{p}$ = 10 nm upon bilayer phosphorene membranes with different twist angles.

Figure 8 shows the residual velocity $V_{r}$ versus initial velocity $V_{0}$ for $r_{p}$ = 10 nm. Similarly to Figure 5, the curves can still be roughly divided into two regions. In region I, the curves are very close to each other. In this region, the relationships between the residual velocity $V_{r}$ and the initial velocity $V_{0}$ are all linear, while the slope of the curves increases almost monotonically as the twist angle increases. This slope increase can be attributed to the increasingly high spikes from reflection and interference of the cone wave as the initial velocity $V_{0}$ increases. In region II, the curves deviate from each other, in which global failure occurs due to reflection and interference of the cone wave. Further, the ballistic limits $V_{50}$ are significantly different from each other for different twist angles. For example, the ballistic limit $V_{50}$ for 0° is 610 m/s, while that for 90° is 440 m/s. More interestingly, there are two types of failure, where one occurs when the projectile bounces back with relatively small $V_{0}$, while the other one occurs when the projectile penetrates the phosphorene membrane.

**Figure 9:**
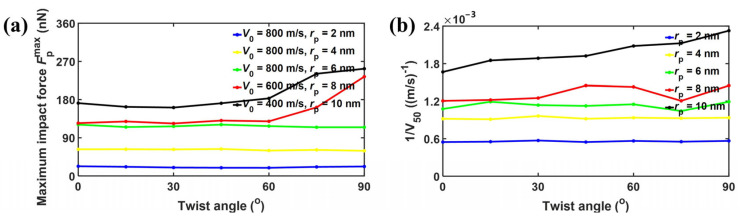
Impact resistance of bilayer phosphorene membranes for projectiles with different $r_{p}$. (**a**) Maximum impact force ${F_{p}}^{max}$ versus twist angle; (**b**) inverse of ballistic limit $1/V_{50}$ versus twist angle.

**Figure 10:**
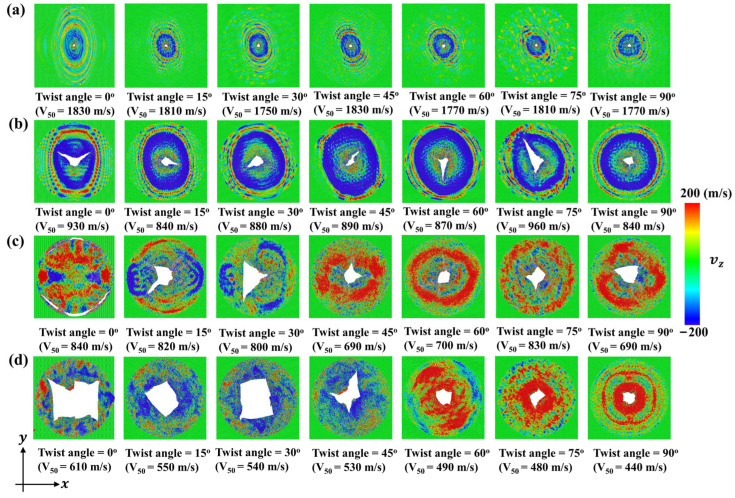
Fracture patterns of the bilayer phosphorene membranes with different twist angles under the impact of the diamond projectiles with different radius $r_{p}$ when the initial velocity $V_{0}$ is equal to the ballistic limit $V_{50}$: (**a**) $r_{p}$ = 2 nm; (**b**) $r_{p}$ = 6 nm; (**c**) $r_{p}$ = 8 nm; (**d**) $r_{p}$ = 10 nm.

Figure 9 shows the impact resistance of bilayer phosphorene membranes for projectiles with different $r_{p}$. For projectiles with $r_{p}$ ≤ 6 nm, the maximum impact force ${F_{p}}^{max}$ barely changes with the twist angle, as shown in Figure 9a. In contrast, for projectiles with $r_{p}$ ≥ 8 nm, the maximum impact force ${F_{p}}^{max}$ first fluctuates for the increase in twist angle from 0° to 60°, and then increases dramatically for the increase in twist angle from 60° to 90°. Figure 9b shows the inverse of the ballistic limit ${1/V}_{50}$ versus the twist angle for visualization and comparison with Figure 9a. It can be seen that for $r_{p}$ ≤ 6 nm, the inverse of the ballistic limit ${1/V}_{50}$ barely changes with the increase in twist angle. As mentioned above, when the projectile radius $r_{p}$ is no bigger than 6 nm, the force amplification is either 0 or smaller than 1.0 from reflection and interference of the cone wave, playing a marginal role in determining the ballistic limit $V_{50}$. In contrast, when the projectile radius $r_{p}$ is no smaller than 8 nm, the force amplification starts to be bigger than 1.0, decreasing the ballistic limit $V_{50}$. Therefore, as shown in Figure 9b, the inverse of ballistic limit ${1/V}_{50}$ increases almost monotonically with the twist angle. In other words, the ballistic limit $V_{50}$ increases as the twist angle increases. Figure 10 shows the fracture patterns of the bilayer phosphorene membranes under the ballistic impact of projectiles with $r_{p}$ from 2 to 10 nm. It can be seen that for $r_{p}$ ≤ 6 nm, the structural failure is local, while the structural failure is global for $r_{p}$ ≥ 8 nm.

## 4. Conclusions

In this work, CG MD simulations were adopted to study the effect of twist angle and projectile radius on the ballistic impact performance of bilayer phosphorene membranes with radius equal to 48 nm. First, the projectile radius was fixed at 4 nm, while the twist angle for the bilayer phosphorene membrane ranges from 0° to 90°. The results indicated that the velocity distribution profiles and propagation of the cone wave are different for membranes with different twist angles. When the twist angle is equal to 0°, the shape of the cone wave front is an ellipse with an axial ratio equal to 1.44. As the twist angle increases from 0° to 90°, the shape of the cone wave front alters, in which the major axis rotates counterclockwise and the axial ratio keeps decreasing to 1.0. Despite the differences in impact dynamics, the twist angle plays a minor role in impact force profile, projectile velocity profile, and the ballistic limit, in which the structural failure is local instead of global. Second, the projectile radius was altered from 2 to 10 nm to investigate its effect on ballistic impact performance. For projectiles with $r_{p}$ ≤ 6 nm, the maximum impact force ${F_{p}}^{max}$ barely changes with the twist angle, while the structural failure is locally induced by the initial collision from the projectile. For projectiles with $r_{p}$ ≥ 8 nm, the maximum impact force ${F_{p}}^{max}$ first fluctuates for the increase in twist angle from 0° to 60° and then increases dramatically for the increase in twist angle from 60° to 90°. Accordingly, the structural failure is global, induced by the reflection and interference of the cone wave. Moreover, due to different degrees of mismatch between the wave-front shape and the membrane shape, the reflection and interference are also different for bilayer phosphorene membranes with different twist angles. Specifically, the aforementioned mismatch for the twist angle equal to 90° is lowest, bringing the maximum force amplification and thus the biggest ballistic limit reduction. For $r_{p}$ = 10 nm, the ballistic limit $V_{50}$ for 0° is 610 m/s, while that for 90° is 440 m/s. Overall, our findings provide timely guidance for the design of future nanodevices using phosphorene with high impact resistance.

## Data Availability

Data will be made available on request.

## Abbreviations

The following abbreviations are used in this manuscript.

2D	two-dimensional
CG	coarse-grained
MD	molecular dynamics
